# Multi-view clustering for single-cell RNA-seq data based on graph fusion

**DOI:** 10.1093/bib/bbaf193

**Published:** 2025-05-25

**Authors:** Jing Wang, Junfeng Xia, Dayu Tan, Yunjie Ma, Yansen Su, Chun-Hou Zheng

**Affiliations:** Anhui Provincial Key Laboratory of Multimodal Cognitive Computation, School of Artificial Intelligence, Anhui University, 111 Jiulong Road, Hefei, 230601, Anhui, China; Institutes of Physical Science and Information Technology, Anhui University, 111 Jiulong Road, Hefei, 230601, Anhui, China; Institutes of Physical Science and Information Technology, Anhui University, 111 Jiulong Road, Hefei, 230601, Anhui, China; School of Computer Science and Information Engineering, Hefei University of Technology, 111 Jiulong Road, Hefei, 230601, Anhui, China; School of Artificial Intelligence, Anhui University, 111 Jiulong Road, Hefei, 230601, Anhui, China; School of Artificial Intelligence, Anhui University, 111 Jiulong Road, Hefei, 230601, Anhui, China

**Keywords:** single-cell RNA-sequencing, multi-view clustering, graph fusion, adaptive learning, rank constraint

## Abstract

Single-cell RNA sequencing (scRNA-seq) provides transcriptome profiling of individual cells, allowing for in-depth studies of cell heterogeneity at cell resolution. While cell clustering lays the basic foundation of scRNA-seq data analysis, the high-dimensionality and frequent dropout events of the data raise great challenges. Although plenty of dedicated clustering methods have been proposed, they often fail to fully explore the underlying data structure. Here, we introduce scMCGF, a new multi-view clustering algorithm based on graph fusion. It utilizes multi-view data generated from transcriptomic data to learn the consistent and complementary information across different view, ultimately constructing a unified graph matrix for robust cell clustering. Specifically, scMCGF utilizes two-dimensional-reduction methods (principal component analysis and diffusion maps) to capture both linear and non-linear characteristics of the data. Additionally, it calculates a cell-pathway score matrix to incorporate pathway-level information. These three features, along with the pre-processed gene expression data, form the multi-view data. scMCGF iteratively refines the structure of similarity graphs of each view through adaptive learning and learns a unified graph matrix by weighting and fusing the individual similarity graph matrix. The final clustering results are obtained by applying the rank constraint on the Laplacian matrix of the unified graph matrix. Experiments results of 13 real data sets reveal that scMCGF outperforms eight state-of-the-art methods in clustering accuracy and robustness. Furthermore, biological analysis validates that the clustering results of scMCGF provide a reliable foundation for downstream investigations.

## Introduction

The rapid development of single-cell RNA sequencing (scRNA-seq) provides comprehensive transcriptome profiling of individual cells, promoting humans to understand and analyze cell heterogeneity and biological systems from a new perspective. Accurate identification of cell types is an important problem in the field of scRNA-seq data analysis, which is of great significance for understanding the essence of life [1–4], the precision treatment of complex diseases (like cancer) [5–7], and research and discovery of drugs [8]. In recent years, although researchers have proposed many unsupervised clustering algorithms dedicated to identifying cell subpopulations from scRNA-seq data [9], the high dimensionality and frequent dropout events underlying the data present significant obstacles to capturing the discriminative features and internal structure of data [10, 11].

Recently, sever graph-based clustering methods have been proposed to distinguish cell subpopulations by exploring structural relationships between cells. For example, SC3 [12] is a consensus clustering method based on multiple cell–cell similarity matrices. Seurat [13] constructs cell-to-cell similarity using shared nearest neighbors and divides the cell population through the Louvain community detection algorithm by maximizing the modularity. SCCLRR [14] captures both global and local intrinsic data properties based on a low-rank representation (LRR) model, and it applies the spectral clustering method [15] to identify new cell types based on the learned similarity matrix. sciPath [16] applies similarity network fusion [17] to integrate pathway-level features and gene-level features to obtain the final fusion similarity matrix, which effectively reduces the impact of noise and improves the performance of clustering.

In parallel, deep learning algorithms based on graph neural networks (GNNs) are also widely used to cluster scRNA-seq data because they can learn the low-dimensional representation of data and the cell–cell relationships by aggregating the information of neighbor nodes. For instance, scGNN [18] utilizes GNN to understand and aggregate cell–cell relationships and models heterogeneous gene expression patterns with a left-truncated mixture Gaussian model. GraphSCC [19] accounts for structural relations between cells with GNN and uses a dual self-supervised module to optimize the representation learned from the GCN and the denoising auto-encoder for better cell clustering. scDSC [20] employs a zero-inflated negative binomial model-based auto-encoder, a GNN module, and a mutual-supervised module to learn node representation and structure features, and it integrates the structure information into a deep clustering algorithm.

Although these methods have achieved some success in scRNA-seq clustering, there are still some problems. They often calculate cell similarity based on distance or similarity measurement, which is inaccurate for high-dimensional data and leads to poorly constructed initial graphs. Besides, the constructed graph structure by the *K*-nearest neighbor remains static through the optimization process, which may seriously affect the algorithm performance. In addition, these algorithms typically extract features from single gene expression data without combining biological context or multi-view information, hindering the improvement of the clustering performance.

To address these issues, we propose a novel multi-view clustering algorithm based on graph fusion called scMCGF. scMCGF learns latent features of cells from multiple perspectives, including pathway analysis and two kinds of dimensional-reduction methods to extract both linear and non-linear features. These learned features, combined with the pre-processed gene expression data, form multi-view data framework, which is further integrated and mined in the subsequent learning process. scMCGF constantly updates the structure of the similarity graph for each view through adaptive learning and learns the unified graph matrix by weighting and fusing the individual similarity graph matrices of each view. The clustering results are obtained by applying a rank constraint on the Laplacian matrix of the unified graph matrix. By integrating multi-view data construction with multi-view fusion learning techniques, scMCGF extracts features from various perspectives and facilitates the simultaneous learning of individual view-specific graph matrix and a unified graph matrix in a mutually reinforcing manner, thereby enhancing the overall effectiveness of clustering. We benchmark scMCGF against eight existing methods on 13 real datasets. Experimental results demonstrate that scMCGF outperforms these methods in clustering accuracy (CA) and algorithm robustness. Additionally, biological analyses verify that the clustering results predicted by scMCGF provide an accurate foundation for downstream analysis.

## Materials and methods

### The framework of scMCGF

**Figure 1:**
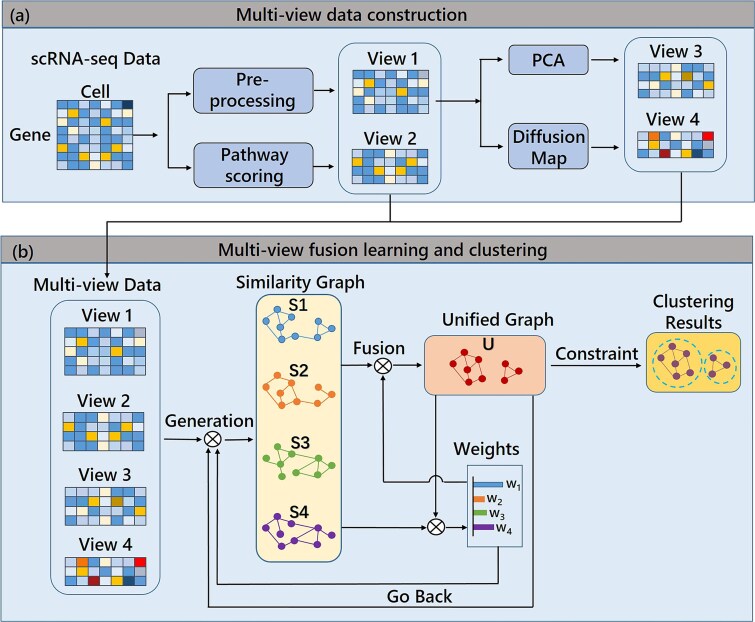
Overview of the scMCGF workflow. (a) Multi-view data construction. We pre-process the raw scRNA-seq data and calculate its pathway score matrix to form two initial data views (View1 and View2). Subsequently, PCA and diffusion maps are utilized to extract linear and nonlinear features, respectively. These extracted features constitute two additional views (View3 and View4). (b) Multi-view fusion learning and clustering. The similarity graphs of each view (S1 ~ S4) and the unified graph (*U*) are learned jointly through adaptive weighted fusion. The final clustering results are obtained by imposing a rank constraint on the Laplacian matrix of the unified graph *U*.

The scMCGF algorithm comprises two main optimization processes: multi-view data construction and multi-view graph fusion learning and clustering (Fig. 1). Specifically, the raw scRNA-seq data is pre-processed and calculated to generate a pathway score matrix, both form two views for scMCGF. The other two views include linear and non-linear features extracted from the pre-processed data by principal component analysis (PCA) and diffusion maps (DMs). Then, scMCGF uses these four views to jointly learn the similarity graph of each view (S1 ~ S4) and a unified graph by adaptive weighted fusion learning. Furthermore, the final clustering results are obtained by applying a rank constraint to the Laplacian matrix of the unified graph (*U*). The details are illustrated in the following sections. The combination of multi-view data can effectively reduce data noise, extract more features beneficial for clustering, and enhance the performance of single-cell clustering. The multi-view fusion technique can automatically assign weight to each data graph matrix to derive the unified graph matrix. This fusion learning method facilitates the learning of each view graph matrix and the unified graph matrix in a mutual reinforcement manner.

### Data sets and multi-view data construction

A total of 13 real scRNA-seq data sets [21–31] are collected from different platforms. Supplementary Table S1 summarizes the details of these data sets. These data sets include various human and mouse tissues, and the sample sizes range from 466 to 34,558. Each cell in these data sets is assigned a category label, which is regarded as a “gold standard” with reference values. To eliminate technical noise and preserve true biological heterogeneity, we pre-process the original scRNA-seq data through the following steps. First, we rescale the data with a log transformation (Base 2) if its range exceeds 100. Second, genes with zero expression values more than 95% of cells are removed. Then we calculate size factors and normalize the data with library size (total real log counts). Suppose that the library size of a specific cell *i* is denoted as *s_i_*, and all cells’ library sizes as *s*, then the size factor of cell *i* is *s_i_*/*s_m_*, where *s_m_* is the median of *s*. Finally, we select the top 2000 highly variable genes from the normalized data as features for subsequent analysis. By removing low expressed or less variable genes, we achieve both computational efficiency and noise reduction. Then the pre-processed data are treated as one view data for the scMCGF algorithm since it retains important information in the data and has an important impact on determining cell types.

A pathway is a collection of relationships between genes that regulate the same biological process [32]. Recent studies have shown that pathway signals extracted from scRNA-seq data can effectively help to classify and cluster heterogeneous cell populations [33]. Since the experimental results in the sciPath paper have already demonstrated the effectiveness of combining *de novo* pathway analysis with clustering methods to improve CA and robustness, we employ the AUCell algorithm to calculate the pathway score of individual cells based on the *de novo* pathway database [35]. This pathway score matrix is then incorporated as another view data in our scMCGF framework. AUCell [34] is an algorithm that scores the activity of each regulon in each cell, which allows researchers to identify cells with active gene sets in scRNA-seq data. When focusing on the pathway gene set, AUCell enables scoring pathways on individual cells based on gene set enrichment analysis. Therefore, we extract the pathway feature information from the scRNA-seq data to capture the relationship among genes and treat it as a view of data in the scMCGF algorithm to improve the clustering performance.

In order to better extract the linear and non-linear features of the scRNA-seq data, scMCGF also utilizes PCA [36] and DMs [37] to reduce the dimensionality of the pre-processed data. The two low-dimensional feature matrices are treated as two additional views of the scMCGF algorithm. We use the Dimensionality Reduction toolbox integrated into the MATLAB environment to realize PCA and DMs. In the scMCGF algorithm, we select the maximum likelihood to estimate the dimensionality of each data set.

### Multi-view similarity graph fusion

To explore the structure information from the constructed multi-view data and learn clustering-beneficially features, scMCGF utilizes sparse representation and adaptive learning to model the learning of the similarity graphs of each view. Specifically, the similarity graph of each view can be constructed by Equation (1) [38]:


(1) \begin{eqnarray*} && \underset{\left\{{S}^v\right\}}{\min}\sum \limits_{v=1}^m\sum \limits_{i,j=1}^n{\left\Vert{x}_i^v-{x}_j^v\right\Vert}_2^2{s}_{ij}^v\nonumber\\&& \quad +\gamma \sum \limits_{v=1}^m\sum \limits_i^n{\left\Vert{s}_i^v\right\Vert}_2^2,\kern1em s.t.\forall v,{s}_{ii}^v=0,{s}_{ij}^v\ge 0,{1}^T{s}_i^v=1 \end{eqnarray*}

where $m$ represents the number of views, ${X}^v=\left\{{x}_1^v,...,{x}_n^v\right\}\in{\mathbb{R}}^{d_v\times n}$ represents the *v*th view feature matrix, ${d}_v$ is the dimensionality of the *v*th view, *n* is the number of cells, ${s}_{ij}^v$ represents the similarity value of sample ${x}_i$ and ${x}_j$ in the *v*th view, and the similarity values between all samples and sample ${x}_i$ form the vector ${s}_i^v$, and $\gamma$ is the parameter that balances the two terms and can be calculated by the model.

Based on these constructed similarity graph matrices, the fusion learning of similarity graphs and the unified graph is realized in a mutual reinforcement manner. Specially, suppose that the unified graph matrix is $U\in{\mathbb{R}}^{n\times n}$, then the fusion learning process is equivalent to solving the following optimization problem [38]:


(2) \begin{equation*} \underset{U}{\min}\sum \limits_{v=1}^m{w}_v{\left\Vert U-{S}^v\right\Vert}_F^2,\kern0.5em s.t.\forall i,{u}_{ij}\ge 0,{1}^T{u}_i=1 \end{equation*}

where ${w}_v$ represents the weight of the *v*th similarity graph matrix, ${u}_i\in{\mathbb{R}}^{n\times 1}$ is a column vector, and ${u}_{ij}$ is the *j*th element of ${u}_i$. According to study [39], the weight ${w}_v$ can be calculated by Equation (3):


(3) \begin{equation*} {w}_v=\frac{1}{2\sqrt{{\left\Vert U-{S}^v\right\Vert}_F^2}} \end{equation*}

Then, combining Equation (1) and Equation (2), the fusion learning process can be coupled into the following optimization problem:


(4) \begin{eqnarray*}&& \underset{\left\{{S}^v\right\},U}{\min}\sum \limits_{v=1}^m\sum \limits_{i,j=1}^n{\left\Vert{x}_i^v-{x}_j^v\right\Vert}_2^2{s}_{ij}^v+\gamma \sum \limits_{v=1}^m\sum \limits_i^n{\left\Vert{s}_i^v\right\Vert}_2^2+\sum \limits_{v=1}^m{w}_v{\left\Vert U-{S}^v\right\Vert}_F^2 \nonumber\\&& {}s.t.\forall v,{s}_{ii}^v=0,{s}_{ij}^v\ge 0,{1}^T{s}_i^v=1,{u}_{ij}\ge 0,{1}^T{u}_i=1 \end{eqnarray*}

### Clustering with constrained Laplacian rank

The similarity graph matrices ${S}^1,{S}^2,...{S}^m$ and the unified graph matrix $U$ can be obtained by iteratively optimizing the Equation (4), and the specific solving process is shown in the section Optimization Algorithms. Since the unified graph matrix $U$ is non-negative, its Laplacian matrix has the following theorem [40, 41].

Theorem 1. *The multiplicity r of the eigenvalue 0 of the Laplacian matrix*  ${L}_U$  *is equal to the number of connected components in the graph of the unified matrix*  $U$, where ${L}_U={D}_U-\left({U}^T+U\right)/2$ is the Laplacian matrix of the unified matrix $U$, and the degree matrix ${D}_U$ is the diagonal matrix whose *i*th diagonal element is ${\sum}_j\left({u}_{ij}+{u}_{ji}\right)/2$.

According to the theorem, if $\operatorname{rank}\left({L}_U\right)=n-c$, then the unified graph $U$ can be partitioned into *c* connected components, which means the data points are partitioned into *c* clusters directly without introducing additional clustering algorithms. Suppose ${\lambda}_i$ represents the *i*th smallest eigenvalue of ${L}_U$*,* and it is noted that ${\lambda}_i\ge 0$ since the unified graph matrix $U$ is positive semi-definite [40], so the constraint $\operatorname{rank}\left({L}_U\right)=n-c$ can be achieved if $\sum \limits_{i=1}^c{\lambda}_i=0$. And according to Ky Fan’s Theorem [42], we have the following formula:


(5) \begin{equation*} \sum \limits_{i=1}^c{\lambda}_i=\underset{F\in{\mathbb{R}}^{n\times c}}{\min } Tr\left({F}^T{L}_SF\right),\kern1em s.t.{F}^TF=I \end{equation*}

where $F=\left[{f}_1,{f}_2,...{f}_c\right]$ is the low-dimensional embedding matrix, and $I\in{\mathbb{R}}^{c\times c}$ is the identity matrix. As a result, the final objective function is:


(6) \begin{align*} & \underset{\left\{{S}^v\right\},U}{\min}\sum \limits_{v=1}^m\sum \limits_{i,j=1}^n{\left\Vert{x}_i^v-{x}_j^v\right\Vert}_2^2{s}_{ij}^v \nonumber\\& \quad +\gamma \sum \limits_{v=1}^m\sum \limits_i^n{\left\Vert{s}_i^v\right\Vert}_2^2+\sum \limits_{v=1}^m{w}_v{\left\Vert U-{S}^v\right\Vert}_F^2+2\lambda Tr\left({F}^T{L}_UF\right) \nonumber\\& {}s.t.\forall v,{s}_{ii}^v=0,{s}_{ij}^v\ge 0,{1}^T{s}_i^v=1,{u}_{ij}\ge 0,{1}^T{u}_i=1,{F}^TF=I \end{align*}

When the parameter $\lambda$ is large enough, the objective function (6) will make $\sum \limits_{i=1}^c{\lambda}_i=0$ hold, which makes the unified graph matrix $U$ contains *c* connected components exactly and partitions the data points into *c* clusters. It is worth stressing that $\lambda$ does not need to be tuned.

### Optimization algorithms

It is hard to solve problem (6) and give each variable an optimized solution since all the variables are coupled together in the objective function and the constraints are not smooth. Here, we solve the problem via alternate iterative optimization and the Augmented Lagrange multiplier scheme [38]. The idea of alternate iterative optimization is that when one variable is updated, the values of the other variables need to be known. Therefore, we begin to solve the problem by initializing the similarity graph matrices ${S}^1,{S}^2,...{S}^m$. According to study [39], the similarity graph matrices ${S}^1,{S}^2,...{S}^m$ can be initialized by solving problem (1), where the value of each element ${s}_{ij}^v$ in ${S}^v$ is as follows:


(7) \begin{equation*} {s}_{ij}^v=\left\{\begin{array}{l}\frac{b_{i,k+1}-{b}_{ij}}{k{b}_{i,k+1}-{\sum}_{h=1}^k{b}_{ih}}\kern1em j\le k\\{}\kern3em 0\kern3em j>k\end{array}\right. \end{equation*}

where ${b}_{ij}={\left\Vert{x}_i^v-{x}_j^v\right\Vert}_2^2$, *k* is the number of neighbors. Similarly, the weight of each view is initialized as ${w}_v=\frac{1}{m}$. According to the initialized ${S}^1,{S}^2,...{S}^m$ and ${w}_v$, $U$, and $F$ can be initialized. The specific updated rules are shown below:

**Fix**  ${w}_v$, $U$, **and**  $F$, **update**  ${S}^1,{S}^2,...{S}^m$: When ${w}_v$, $U$, and $F$ are fixed, the last term of problem (6) is a constant. Updating ${S}^1,{S}^2,...{S}^m$ is to solve the following problem:


(8) \begin{align*} & \underset{\left\{{S}^v\right\}}{\min}\sum \limits_{v=1}^m\sum \limits_{i,j=1}^n{\left\Vert{x}_i^v-{x}_j^v\right\Vert}_2^2{s}_{ij}^v+\gamma \sum \limits_{v=1}^m\sum \limits_i^n{\left\Vert{s}_i^v\right\Vert}_2^2+\sum \limits_{v=1}^m{w}_v{\left\Vert U-{S}^v\right\Vert}_F^2 \nonumber\\ & \quad{}s.t.\forall v,{s}_{ii}^v=0,{s}_{ij}^v\ge 0,{1}^T{s}_i^v={1}_{\circ} \end{align*}

According to formula (7), the calculation of ${S}^v$ for each view is independent. Thus, we can update ${S}^v$ one by one through optimizing the following objective function:


(9) \begin{align*} & \underset{S^v}{\min}\sum \limits_{i,j=1}^n{\left\Vert{x}_i^v-{x}_j^v\right\Vert}_2^2{s}_{ij}^v+\gamma \sum \limits_i^n{\left\Vert{s}_i^v\right\Vert}_2^2+{w}_v{\left\Vert U-{S}^v\right\Vert}_F^2\nonumber\\& \quad{}s.t.{s}_{ii}^v=0,{s}_{ij}^v\ge 0,{1}^T{s}_i^v={1}_{\circ} \end{align*}

In practice, a cell prefers to be related only to its neighbors. Therefore, according to study [39], ${s}_{ij}^v$ in ${S}^v$ with *k* non-zero values can be calculated by Equation (10):


(10) \begin{equation*} {s}_{ij}^v=\left\{\begin{array}{l}\frac{e_{i,k+1}-{e}_{ij}+2{w}_v{u}_{ij}-2{w}_v{u}_{i,k+1}}{k{e}_{i,k+1}-{\sum}_{h=1}^k{e}_{ih}-2k{w}_v{u}_{i,k+1}+2{\sum}_{h=1}^k{w}_v{u}_{ih}}\kern1em j\le k\\{}\kern8em 0\kern6.36em j>k\end{array}\right. \end{equation*}

where ${e}_{ij}={\left\Vert{x}_i^v-{x}_j^v\right\Vert}_2^2$.

**Fix**  ${S}^1,{S}^2,...{S}^m$, $U$, **and**  $F$  **update**  ${w}_v$: When ${S}^1,{S}^2,...{S}^m$, $U$, and $F$ are fixed, optimizing problem (6) is equivalent to solving problem (2). The value of ${w}_v$ can be formulated as Equation (3):


(3) \begin{equation*} {w}_v=\frac{1}{2\sqrt{{\left\Vert U-{S}^v\right\Vert}_F^2}} \end{equation*}

**Fix**  ${S}^1,{S}^2,...{S}^m$, ${w}_v$, **and**  $F$  **update**  $U$: When ${S}^1,{S}^2,...{S}^m$, ${w}_v$, and $F$ are fixed, since $Tr\left({F}^T{L}_UF\right)=\frac{1}{2}{\sum}_{i,j}{\left\Vert{f}_i-{f}_j\right\Vert}_2^2{u}_{ij}$, the optimization of problem (5) can be transformed into:


(11) \begin{equation*} {\displaystyle \begin{array}{l}\underset{u^i}{\min}\sum \limits_{v=1}^m\sum \limits_{j=1}^n{w}_v{\left({u}_{ij}-{s}_{ij}^v\right)}^2+\lambda \sum \limits_{j=1}^n{\left\Vert{f}_i-{f}_j\right\Vert}_2^2{u}_{ij}\\{}s.t.\forall i,{u}_{ij}\ge 0,{1}^T{u}_i=1\end{array}} \end{equation*}

Since each cell is independent, the problem can be solved separately for each cell as follows:


(12) \begin{equation*} {\displaystyle \begin{array}{l}\underset{u^i}{\min}\sum \limits_{v=1}^m\sum \limits_{j=1}^n{w}_v{\left({u}_{ij}-{s}_{ij}^v\right)}^2+\lambda \sum \limits_{j=1}^n{\left\Vert{f}_i-{f}_j\right\Vert}_2^2{u}_{ij}\\{}s.t.\forall i,{u}_{ij}\ge 0,{1}^T{u}_i=1\end{array}} \end{equation*}

Denote ${d}_{ij}={\left\Vert{f}_i-{f}_j\right\Vert}_2^2$, which represents the *j*th element of ${d}_i$, and similarly for ${u}_i$ and ${s}_i$. Thus, Equation (12) can be rewritten as:


(13) \begin{equation*} \underset{u_i}{\min}\sum \limits_{v=1}^m{\left\Vert{u}_i-{s}_i^v+\frac{\lambda }{2m{w}_v}{d}_i\right\Vert}_2^2,\kern1em s.t.\forall i,{u}_{ij}\ge 0,{1}^T{u}_i=1 \end{equation*}

**Figure 2:**
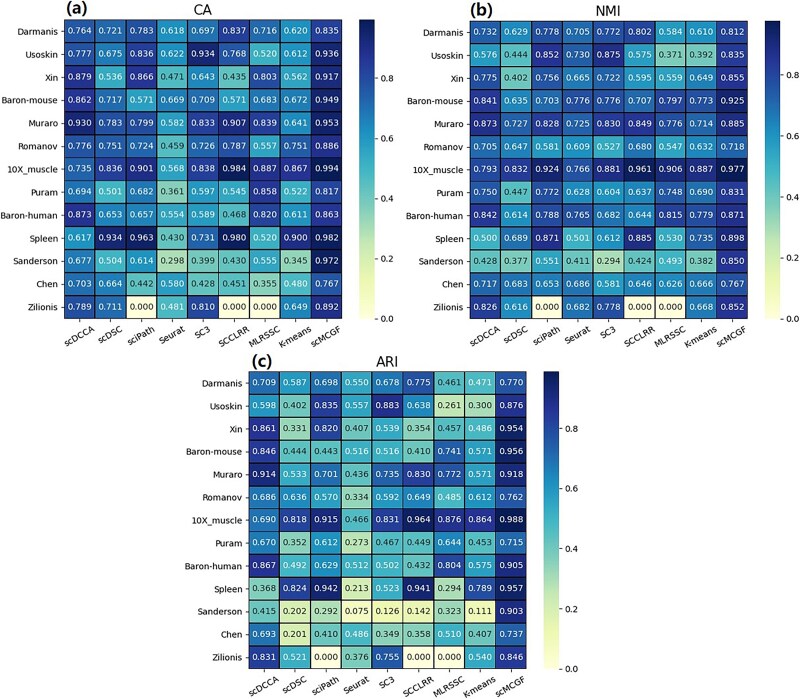
Benchmarking scMCGF against other methods in 13 scRNA-seq data sets. Clustering performance comparison of scDCCA, scDSC, sciPath, Seurat, SC3, SCCLRR, MLRSSC, scMCGF, and *K*-means measured by (a) CA, (b) NMI, and (c) ARI.

According to study [38], the optimal solution of ${u}_i^{\ast }$ can be solved by the following equation:


(14) \begin{equation*} {u}_i^{\ast }=\frac{\sum_{v=1}^m{q}^v}{m}+\frac{1}{n}-\frac{\sum_{v=1}^m{1}^T{q}^v1}{mn}-\frac{1^T{\varphi}^{\ast }1}{mn}+\frac{\varphi^{\ast }}{m} \end{equation*}

where ${q}^v={s}_i^v-\frac{\lambda }{2m{w}_v}{d}_i$. Suppose that $p=\frac{\sum_{v=1}^m{q}^v}{m}+\frac{1}{n}-\frac{\sum_{v=1}^m{1}^T{q}^v1}{mn},{\hat{\varphi}}^{\ast }=\frac{1^T{\varphi}^{\ast }}{mn}$, then ${u}_i^{\ast }=p-{\hat{\varphi}}^{\ast }1+\frac{\varphi^{\ast }}{m}$. For any *j*, there is


(15) \begin{equation*} {u}_{ij}^{\ast }={p}_j-{\hat{\varphi}}^{\ast }+\frac{\varphi_j^{\ast }}{m}={\left({p}_j-{\hat{\varphi}}^{\ast}\right)}_{+} \end{equation*}

where ${(a)}_{+}=\max \left(a,0\right)$, let $f\left(\overset{\wedge }{\varphi}\right)=\frac{1}{n}\sum \limits_{j=1}^n{\left(\hat{\varphi}-{p}_j\right)}_{+}-\hat{\varphi}$, then ${\hat{\varphi}}^{\ast }$ can be calculated by solving the root finding problem as $f\left({\hat{\varphi}}^{\ast}\right)=0$. The root of $f\left(\hat{\varphi}\right)=0$ can be solved via the Newton method as follows:


(16) \begin{equation*} {\hat{\varphi}}_{t+1}={\hat{\varphi}}_t-\frac{f\left({\hat{\varphi}}_t\right)}{f^{\hbox{'}}\!\left({\hat{\varphi}}_t\right)} \end{equation*}

**Fix**  ${S}^1,{S}^2,...{S}^m$, ${w}_v$, **and**  $U$  **update**  $F$: When ${S}^1,{S}^2,...{S}^m$, ${w}_v$, and $U$ are fixed, optimizing problem (6) is equivalent to solving problem (17):


(17) \begin{equation*} \underset{F}{\min } Tr\left({F}^T{L}_UF\right),s.t.{F}^TF=I \end{equation*}

According to the aforementioned iterative optimization process, all the variables can be updated. By repeating the iterative process until the rank constraint is satisfied or the maximum iteration is reached, the unified graph $U$ with *c* connected components and the final clustering result can be obtained.

**Figure 3:**
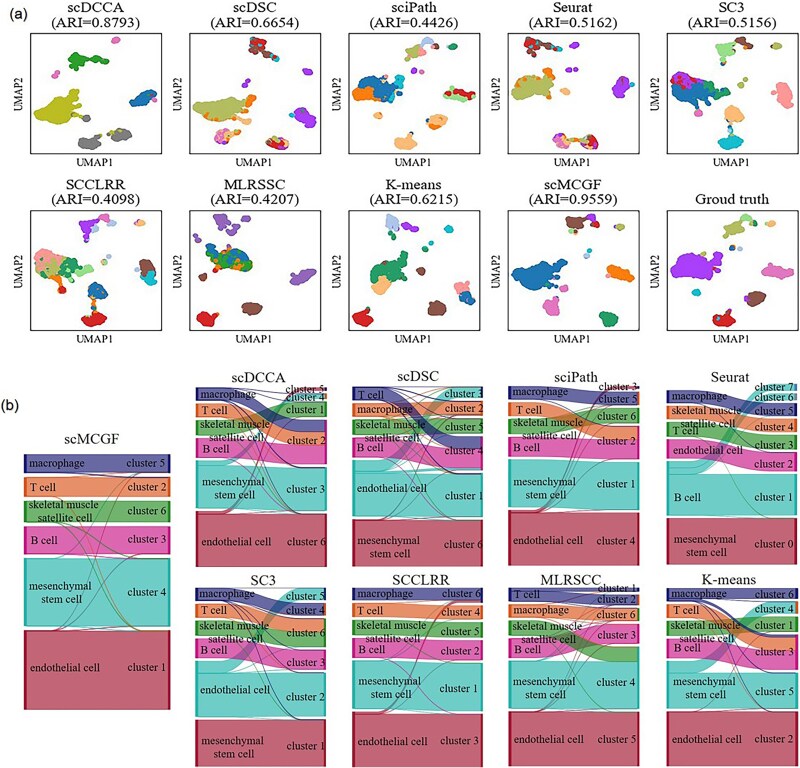
Cell visualization and Sankey diagrams. (a) Cell visualization of the identified clusters by nine methods and the ground truth on Baron-mouse data sets. (b) Sankey plots of clustering results by scMCGF and eight other comparison methods for the Qx_limb_muscle data set.

### Clustering metrics

Three commonly used clustering metrics are used to evaluate the clustering performance of different methods, including CA [43], normalized mutual information (NMI) [44], and adjusted rand index (ARI) [45]. The definitions of these three metrics can be found in Section A of the Supplementary file. Both CA and NMI produce scores from 0 to 1, and the score of ARI ranges from −1 to 1. Higher values represent better clustering performances.

## Results and discussion

### Performance comparison with other methods

To assess the clustering performance of scMCGF, we apply it to 13 real scRNA-seq data sets and compare it with eight state-of-the-art methods, including scDCCA, scDSC, sciPath, Seurat, SC3, SCCLRR, MLRSSC [46], and *K*-means. Among them, scDCCA and scDSC are deep learning-based scRNA-seq clustering methods based on contrastive learning and GNN. SC3, Seurat, and sciPath are scRNA-seq clustering methods based on cell similarity. SCCLRR captures features of scRNA-seq data based on a LRR model. Although MLRSSC is not designed for scRNA-seq data, it is applicable when treating different data sources as different data views. *K*-means is a base clustering method that performs clustering without prior information. All algorithms are executed with default parameters, and the number of clusters for each data sets are determined based on its ground truth. And three metrics (CA, NMI, and ARI) are employed to evaluate clustering performance. For scDCCA and scDSC, experiments are repeated 10 times to ensure statistical significance. For sciPath, we select SC3 as the clustering algorithm of sciPath for data sets with cell numbers less than 5000, while Seurat for data sets with more than 5000 cells.

Figure 2 shows the heat maps of three metrics values of these nine algorithms in 13 real scRNA-seq data sets. The results indicate that scMCGF achieves the best clustering performance. Specifically, scMCGF outperforms the other eight clustering algorithms in 12 data sets (except Chen) with all CA values higher than 0.8, indicating its excellent generalizability. In addition, scMCGF achieves the optimal CA values on 10 of these data sets. Although it does not achieve the highest CA values on Darmanis, Puram, and Baron-human data sets, it still performs second best. Similarly, according to NMI and ARI, scMCGF ranks first on 12 data sets except for Usoskin. For Usoskin, the NMI and ARI values of scMCGF are only lower than those of the optimal algorithm by 4.06% and 0.6%, respectively. Furthermore, scMCGF performs significantly better than the other eight methods on some data sets. For example, for the Sanderson data set, the NMI and ARI values of scMCGF are, respectively, higher than those of the second-best methods (sciPath and Seurat) by 29.9% and 48.79%, indicating that scMCGF can achieve excellent performance on complex data sets.

**Figure 4:**
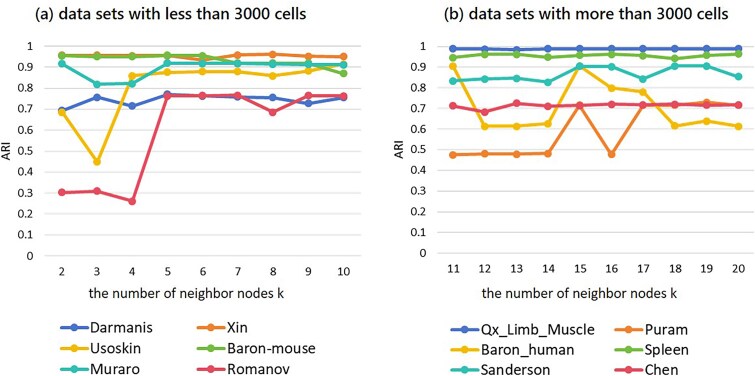
Clustering performance (ARI) of scMCGF on 12 data sets with different parameter *k*. (a) Six data sets with less than 3000 cells and (b) six data sets with more than 3000 cells.

To demonstrate the intuitive clustering effect of these algorithms, we select two real data sets (Baron-mouse and Qx_limb_muscle) to perform cell visualization and plot the Sankey diagram. The cell visualization and Sankey plots show the difference between the clustering results of each method and the true labels. Specifically, for Baron-mouse, we apply UMAP [47] to project the pre-processed gene expression data into the two-dimensional (2D) space based on the predicted cell clusters of each method and the ground truth labels. As shown in Fig. 3a, each point denotes a cell, and different colors mean different cell clusters. The cell visualization of sciPath, SC3, SCCLRR, and MLRSSC mix different cell subtypes, leading to errors. Although scDCCA achieves high CA (ARI = 0.8793), it still suffers from the same challenges as other methods, such as merging smaller cell clusters into a single whole. In contrast, the boundaries of various cell clusters predicted by scMCGF are clearer than those of the other methods, and cell clusters predicted by scMCGF are closer to ground truth, implying that scMCGF is superior to other competing methods. For Qx_Limb_Muscle, we plot its Sankey diagrams based on the cell labels predicted by each method and the ground truth. As shown in Fig. 3b, Seurat incorrectly divided the larger B cell population into multiple clusters, while scDCCA, scDSC, sciPath, SC3, and *K*-means are prone to merge different clusters into the same one. Although SCCLRR achieves high clustering performance (ARI = 0.8793), it still misclassifies some endothelial cells into macrophage clusters. By comparison, scMCGF achieves the best clustering results, showing clearer cell clusters and higher consistency with the ground truth.

### Parameter analysis

The number of the neighbor nodes *k* is an important parameter in our scMCGF algorithm. To investigate its influence on the clustering performance of each data set, we run our model on 12 datasets with the parameter at different *k* values. Specifically, for data sets with less than 3000 cells, *k* is limited to the range [2–10], and for data sets with more than 3000 cells, *k* is restricted to the range [11–20]. Figure 4 shows the clustering performance (ARI) of scMCGF on 12 scRNA-seq data sets with different *k*. The results indicate that for data sets with less than 3000 cells, when *k* < 5, the ARI values of four data sets (except Xin and Baron-mouse) are not optimal and highly variable; while when *k* > 5, the ARI values of four data sets (except Baron-mouse and Romanov) tends to be stable. Therefore, *k* = 5 is the optimal choice according to the clustering performance of these six data sets. Similarly, for data sets with more than 3000 cells, the ARI values of the Qx_Limb_Muscle, Spleen, and Chen data sets change little at different *k* values, indicating that the three data sets are insensitive to this parameter. Another three data sets Puram, Baron-human, and Spleen achieve the optimal clustering performance at *k* = 15. Therefore, considering the clustering performance and computation timeliness, we select *k* = 5 as the optimal number of neighbor nodes for data sets with less than 3000 cells, while *k* = 15 as the optimal parameter for data sets with more than 3000 cells.

**Figure 5:**
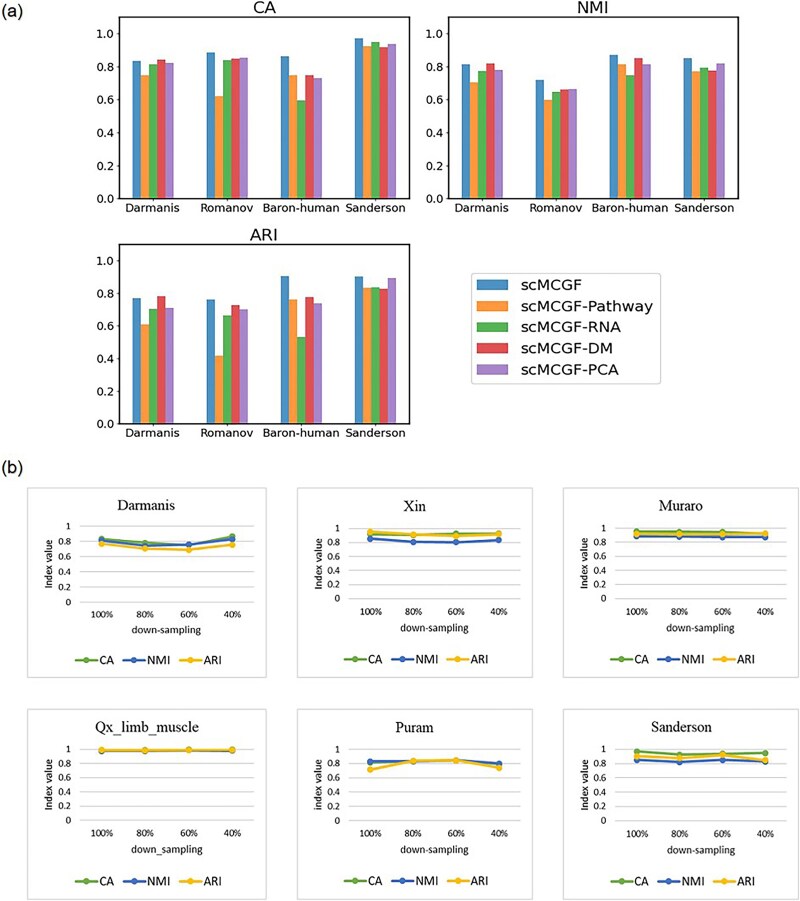
Results of ablation and robustness experiments. (a) Clustering performance comparison of scMCGF and its four variants. scMCGF pathway (without cell pathway scoring data), scMCGF-RNA (without pre-processed expression data), scMCGF-DM (without feature data extracted by diffusion maps), and scMCGF-PCA (without feature data extracted by PCA). (b) Robustness experiment on six data sets. The figure shows the changes in three metrics values when the data is down-sampled to 80%, 60%, and 40% of the original data set size.

### Ablation study

In this section, we assess the contribution of multi-view data to the clustering performance of our scMCGF algorithm. For the convenience of analysis, we remove each data view to obtain four variants of scMCGF, namely, scMCGF-pathway (without cell pathway scoring data) and scMCGF-RNA (without pre-processed expression data), scMCGF-DM (without feature data extracted by DMs), and scMCGF-PCA (without feature data extracted by PCA). We apply four data sets with different species and sample sizes (Darmanis, Romanov, Baron-Human, and Sanderson) to perform the ablation experiments. For fairness of the comparison, the parameters of the four variants are the same as those of scMCGF, and three clustering metrics (CA, NMI, and ARI) are employed to evaluate the clustering performance of scMCGF and its variants. The results (Fig. 5a and Supplementary Table S6) indicate that the clustering performance of scMCGF is superior to its four variants on three data sets except Darmanis. The clustering performance of scMCGF on Darmanis is very close to the optimal performance achieved by the variant scMCGF-DM. These results indicate that scMCGF outperforms its four variants and the essential role of each view of data. For example, on the Baron-human data set, scMCGF significantly increased by 14.40%, 37.33%, 12.84%, and 16.78% compared with its four variants (scMCGF-pathway, scMCGF-RNA, scMCGF-DM, and scMCGF-PCA) as per the ARI, respectively.

**Figure 6:**
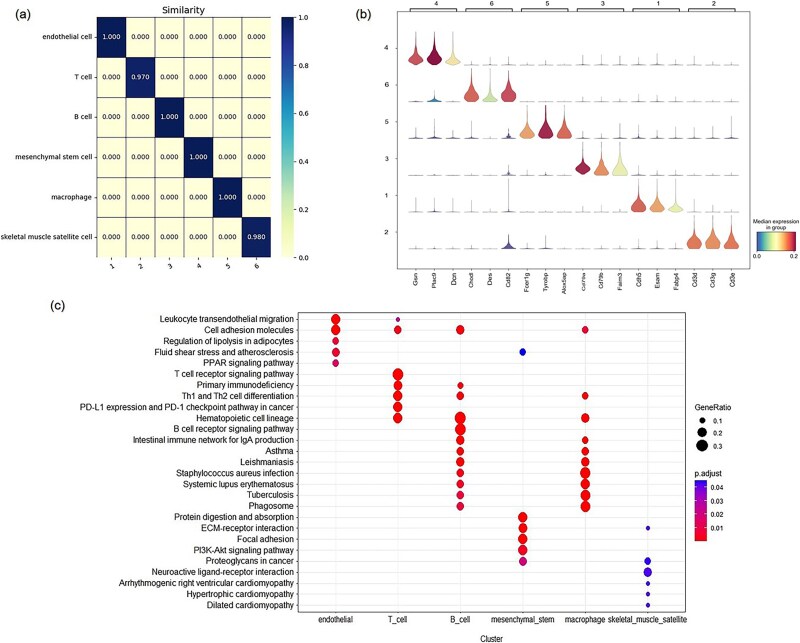
Biological analysis on Qx_limb_muscle data set. (a) Similarity of DEGs between cell clusters and cell types. (b) The KEGG pathway enrichment of the top 50 DEGs across different cell types of the Qx_limb_muscle data set. The gene ratio represents the proportion of DEGs associated with a pathway (higher values indicate more DEGs linked to the pathway). The adjusted *P*-value reflects the statistical significance of the association (smaller values indicate a strong likelihood of the pathway being relevant to the cell subpopulation). (c) Gene expression violin plots of the top three marker genes identified by COSG for each cell type.

### Robustness analysis

To verify the robustness of scMCGF, six real scRNA-seq data sets with different sample sizes are selected to implement the down-sampling experiment. These data sets encompass Darmanis, Xin, Muraro, Qx_limb_muscle, Puram, and Sanderson. Each data set is randomly down-sampled to obtain partial data sets containing 80%, 60%, and 40% cells, respectively. We execute pre-processing, dimensionality reduction, and AUCell score matrix construction of cell pathways according to the method described in Section Data sets and multi-view data construction on these down-sampled data. Then, the obtained multi-view data are input for scMCGF testing, using the same parameters as the whole data to ensure consistency. Three clustering metrics (CA, NMI, and ARI) are adopted to evaluate the clustering performance of scMCGF on these data sets. The experimental results (Fig. 5b and Supplementary Table S7) show that the clustering performance of scMCGF on the down-sampled data has little difference from the complete data, indicating that our scMCGF is still stable and robust when dealing with incomplete data.

**Figure 7:**
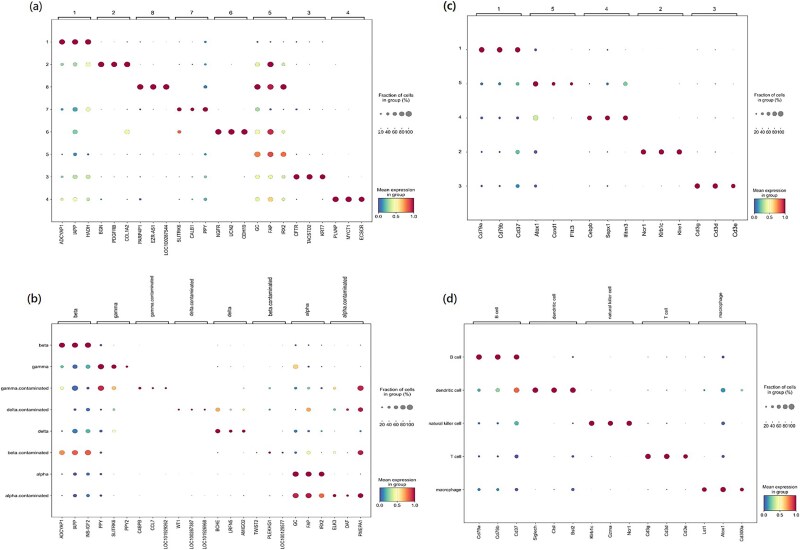
Expression dot plots of the top three marker genes identified COSG. (a) Top three marker genes for predicted cell clusters by scMCGF on Xin data set, (b) top three marker genes for true cell types on Xin data set, (c) top three marker genes for predicted cell cluster by scMCGF on Spleen data set, and (d) top three marker genes for true cell types on Spleen data set.

### Biological analysis

To verify the CA of scMCGF on scRNA-seq data and explore whether these clustering results are conducive to downstream biological analysis, we select Qx_limb_muscle to perform cell annotation, Kyoto Encyclopedia of Genes and Genomes (KEGG) enrichment analysis, and marker gene identification. Specifically, for Qx_limb_muscle, according to the clustering results of scMCGF and ground truth, we find the top 50 differential expressed genes (DEGs) of each cluster and each cell type with COSG package [48] and annotate cells by comparing the similarities between the DEGs of predicted cell clusters and those of real cell types. We calculate the similarity by dividing the number of overlapping DEGs by 50. The DEGs similarity heat map of Qx_limb_muscle is shown in Fig. 6a. It can be seen that for each cell type, there is a cell cluster with the highest similarity to its DEGs, and the highest similarity exceeds 0.95. Therefore, we can annotate each cell cluster to the cell type with the highest similarity. For example, the predicted cell clusters 1–6 are annotated as endothelial cells, T cells, B cells, mesenchymal stem cells, macrophages, and skeletal muscle satellite cells, respectively.

To further explore the functional role of each cell type and verify the biological significance of these DEGs, we perform a KEGG enrichment analysis of these DEGs. Figure 6b shows the KEGG enrichment results of the top 50 DEGs of each subpopulation. It can be seen that the DEGs of different cell types are significantly enriched in diverse pathways, suggesting that they differ not only in expression patterns but also in the biological pathways. And the biological enrichment pathways are also verifiable. For example, the top 50 DEGs of B cells in the Qx_limb_muscle data set are significantly enriched in pathways such as “Hematopoietic cell lineage” and “B cell receptor signaling pathway.” This enrichment underscores the potentially vital role of B cells in the two biological pathways, a finding that aligns with previous studies [49].

We also use COSG to identify the marker genes of each cell cluster predicted by scMCGF on the Qx_limb_muscle data set. Figure 6c shows the expression violin plots of the top three marker genes identified by COSG for each cluster. As shown, the marker genes exhibit distinct expression profiles unique to their respective clusters. To further verify the consistency of these marker genes with ground-truth annotations, we compare them with marker genes reported in PanglaoDB [50] database. The results show that a partial of the identified marker genes are consistent with those recorded in PanglaoDB database. For instance, three marker genes (CD3D, CD3E, and CD3G) of T cells identified by COSG based on predicted cell labels of scMCGF are also documented in the database. “Marker genes” identified by COSG while not listed in PanglaoDB database may represent potential novel marker genes worthy of further investigation. Furthermore, we select two more data sets (Xin and Baron_mouse) and use the COSG package to identify marker genes for both the predicted cell clusters and the true cell types, and then compare the corresponding results. Figure 7 shows the top three marker genes identified by COSG for each cluster predicted by scMCGF and for each true cell types on two data sets (Xin and Baron_mouse). Specifically, Fig. 7a and b shows the top three marker genes for the predicted cell clusters and the true cell types in the Xin data set, respectively. Similarly, Fig. 7c and d corresponds to the Spleen data set. The results demonstrate a high degree of consistency between marker genes identified by COSG for the predicted cell clusters and those for the true cell clusters. For example, in the Xin data set, ADCYAP1, and IAPP are identified in both cell cluster 1 and beta cell type, and PPY and SLITRK6 are identified in cell cluster 7 and gamma cell type, respectively. Similar observations are made in the Spleen data set, as shown in Fig. 7c and d. This consistency indicates that the cell clusters predicted by our model closely match true cell types, which is conducive to the identification of marker genes.

In summary, the distinct patterns of differential gene expression, along with the verifiability of biological enrichment pathways and marker genes confirm the reliability of cell annotation. These biological analyses indicate that the accurate clustering results predicted by scMGCF are beneficial for scRNA-seq downstream analysis, which is also significant in understanding of disease mechanisms and potential treatments.

## Conclusion

The rapid evolution and widespread of single-cell sequencing technology promote the development of computational methods for analyzing single-cell transcriptome data. Among these, graph-based cell clustering methods have gained prominence for their ability to learn the heterogeneous relationships and complex structures within the data. Although they have improved the accuracy of cell clustering, limitations such as focusing exclusively on the similarity of cells at the level of gene expression, neglecting biological context, failing to mine data structure from multiple perspectives, and relying on fixed graph structure in the learning process still exist. These shortcomings significantly affect the clustering performance.

In this study, we propose scMCGF, a new multi-view clustering algorithm based on graph fusion for scRNA-seq data. scMCGF extracts different low-dimensional features and pathway features of gene expression data to generate multi-view data, and it constantly updates and learns the similarity graphs of each view and the unified graph matrix through adaptive learning and automated weighting fusion. By imposing the rank constraint on the Laplacian matrix of the unified graph matrix, scMCGF obtains cell clusters without additional clustering algorithms. Experimental results across 13 real datasets reveal that scMCGF outperforms eight state-of-the-art methods in CA and algorithm robustness. Biological analyses, including cell-type annotation, marker gene identification, and pathway enrichment, validate that clustering results predicted by scMCGF provide an accurate basis for downstream analysis.

Although scMCGF provides a new perspective for analyzing scRNA-seq data, it is not without limitations. For example, the computational efficiency of the algorithm requires improvement. It is worth trying to enhance the speed and scalability of multi-view clustering by exploring parallel computing. Furthermore, the application scope of scMCGF can be expanded beyond scRNA-seq data. Future work could involve applying scMCGF to single-cell multi-omics data analysis, such as stain accessibility analysis and single-cell DNA methylation data analysis.

**Key Points**

- We propose a novel multi-view clustering algorithm called scMCGF based on graph fusion for single-cell RNA-seq data. scMCGF leverages diverse characteristics from transcriptomic data to construct multi-view representations, and learns consistent and complementary information across these views to enhance the accuracy of cell-type identification.
- scMCGF utilizes two dimensionality reduction methods (principal component analysis and diffusion maps) to capture both linear and non-linear characteristics of the transcriptomic data, respectively. Additionally, it learns the characteristics of the cell in the pathway perspective by calculating a cell-pathway score matrix, which helps to reduce noise and improve clustering performance.
- By treating the preprocessed transcriptomic data, two low-dimensional embedding features, and the cell-pathway score of cells as four distinct views, scMCGF constantly learns and updates the similarity graphs of each view and the unified graph through an adaptive and automatic weighted fusion way.
- Extensive experiments demonstrate that scMCGF outperforms eight state-of-the-art methods in clustering accuracy and algorithm robustness. Biological analyses further confirm that the clustering results of scMCGF provide a reliable foundation for downstream analysis.

## Supplementary Material

Supplementary_materials_of_scMCGF_bbaf193

## Data Availability

All data sets analyzed in this paper are publicly available. Specifically, Sanderson and Zilionis originate from the Broad Institute Single Cell Portal, Puram is downloaded from Gene Expression Omnibus, and Spleen, and Qx_Limb_Muscle are available at https://github.com/xuebaliang/scziDesk. The remaining eight data sets come from the website https://hemberglab.github.io/scRNA.seq.datasets.

## Code availability

The source code is available at https://github.com/WJ319/scMCGF.
