# Supplementary material for: Multi-view clustering for single-cell RNA-seq data based on graph fusion
Source: Brief Bioinform. 2025 May 25;26(3):bbaf193. doi: 10.1093/bib/bbaf193 (PMC12103903; doi:10.1093/bib/bbaf193)
Supplement: Supplementary_materials_of_scMCGF_bbaf193 [file supplementary_materials_of_scmcgf_bbaf193.docx]

Supplementary materials of “scMCGF: Multi-view clustering for single-cell RNA-seq data based on graph fusion”

Jing Wang^1^, Junfeng Xia^2^, Dayu Tan^2^, Yunjie Ma^3^, Yansen Su^4^*, and Chunhou Zheng^4^*

1. Anhui Provincial Key Laboratory of Multimodal Cognitive Computation, School of Artificial Intelligence, Anhui University, Hefei, China;
2. Institutes of Physical Science and Information Technology, Anhui University, Hefei 230601, China;
3. School of Computer Science and Information Engineering, Hefei University of Technology, Anhui, China
4. School of Artificial Intelligence, Anhui University, Hefei 230601, China.

**Contents**

1. **Supplementary Methods**
2. Clustering Internal Indexes
3. The identification of DEGs by COSG
4. **Supplementary Tables**

Table S1. The scRNA-seq datasets used in this study

Table S2. Clustering performance accessed by CA on 13 scRNA-seq data sets.

Table S3. Clustering performance accessed by NMI on 13 scRNA-seq data sets.

Table S4. Clustering performance accessed by ARI on 13 scRNA-seq data sets.

Table S5. ARI values of scMCGF on 12 data sets with different parameter k.(A) Six data sets with less than 3000 cells; (B) Six data sets with more than 3000 cells.

Table S6. Performance comparison of scMGCF and its four variants.

Table S7. Clustering performance of scMCGF on whole data and down-sampling data.

1. **Supplementary Methods**
2. **Clustering external Indexes**

**Clustering Accuracy**

Clustering accuracy (CA) represents the proportion of the predicted labels obtained by clustering in the real labels of data. It can be defined as:

$ACC=\max_{m} \frac{\sum_{i=1}^{n} 1\left\{l_{i}=m(c_{i})\right\}}{n}$ (1)

where $l_{i}$ is the ground-truth label, $c_{i}$ is the cluster assignment produced by the algorithm, and $m$ ranges over all possible one-to-one mappings between clusters and labels.

**Normalized Mutual Information**

Normalized Mutual information (NMI) is a commonly used index to measure the quality of the clustering result. It detects the accuracy of the clustering result by calculating the difference ratio between the clustering result and the true partition the degree of correlation between two sets through the entropy of clustering labels and predictive labels. Suppose there are $N$ samples, given the two clustering assignments $U$ and $V$ on the set. $U$ and $V$are prediction labels and true labels, respectively. Then NMI is defined as follows

$NMI=\frac{\sum_{p=1}^{C_{U}} \sum_{q=1}^{C_{V}} \left|U_{p}\cap V_{q}\right|\log\frac{n\left|U_{p}\cap V_{q}\right|}{\left|U_{p}\right|\times\left|V_{q}\right|}}{\max(-\sum_{p=1}^{C_{U}} \left|U_{p}\right|\log\frac{\left|U_{p}\right|}{n},-\sum_{q=1}^{C_{V}} \left|V_{q}\right|\log\frac{\left|V_{q}\right|}{n})}$ (2)

**Adjust Rand Index**

The Rand Index (RI) [35] can calculate the similarity between two clusters by label pairs assigned to the same or different clusters in the real cluster and predicted cluster. ARI is an improved version of RI, which corrects for the lack of a constant value of the Rand index when the cluster assignments are selected randomly. It can be formally defined as

$ARI=\frac{(_{2}^{n})(a+d)-[(a+b)(a+c)+(c+d)(b+d)]}{(_{2}^{n})-[(a+b)(a+c)+(c+d)(b+d)]}$ (3)

where, a is defined as the number of pairs of two objects in the same group in both $U$ and $V$, and b represents the number of pairs of two objects in different groups in both $U$ and $V$, and c represents the number of pairs of two objects in the same group in $U$ but in different groups in $V$, while d is the number of pairs of two objects in different groups in $U$ but in the same group in $V$.

1. **The identification of DEGs by COSG**

COSG uses cosine similarity to evaluate the expression specificity of genes, and the cosine similarity of two genes equals the cosine value of the angle between the two genes’ representative vectors in the cell space. Specifically, to identify marker genes for each cell group, COSG first creates an artificial gene ($\lambda_{k}$) which only expresses in cells of a given group, e.g. Group k ($G_{k}$) and does not express in any other cell groups, thus $\lambda_{k}$is regarded as the idea marker gene for cells belong to $G_{k}$. Then the representative vector for each expressed gene ($g_{i}$) will be compared with the representative vector $\lambda_{k}$, and genes whose representative vectors form the smallest angles with the representative vector of $\lambda_{k}$ and the largest angles with the representative vector of other cell groups will be selected as the marker genes for $G_{k}$. COSG defines a score to evaluate whether $g_{i}$ is a good marker gene for group $G_{k}$. The score is defined as follows:

$COSGscore(g_{i},G_{k})=\cos(g_{i},\lambda_{k})*\frac{\cos(g_{i},\lambda_{k})^{2}}{\cos(g_{i},\lambda_{k})^{2}+\mu\times\sum_{t\in\{1,...K\},t\neq k} \cos(g_{i},\lambda_{t})^{2}}$ (7)

where the term $\cos(g_{i},\lambda_{k})$ represents the cosine similarity between any detected gene $g_{i}$ and the ideal marker gene $\lambda_{k}$, and the term $\frac{\cos(g_{i},\lambda_{k})^{2}}{\cos(g_{i},\lambda_{k})^{2}+\mu\times\sum_{t\in\{1,...K\},t\neq k} \cos(g_{i},\lambda_{t})^{2}}$ is used as the penalty coefficient, and by default, COSG sets $\mu=1$. Based on this score, COSG can output a list of candidate marker genes starting with the ones with the highest COSG scores for each cell group. In this paper, the top 50 candidate marker genes of each group are selected as its DEGs.

**2. Supplementary Tables**

**Table S1.** The scRNA-seq data sets used in this study.

| Data set | Cells | Genes | Cell Types | Cell Source | Reference |
| --- | --- | --- | --- | --- | --- |
| Darmanis | 466 | 22088 | 9 | Human brain | [1] |
| Usoskin | 622 | 25334 | 4 | Mouse brain | [2] |
| Xin | 1600 | 39851 | 8 | Human pancreas | [3] |
| Baron-mouse | 1886 | 14878 | 13 | Mouse pancreas | [4] |
| Muraro | 2126 | 19127 | 10 | Human pancreas | [5] |
| Romanov | 2881 | 24341 | 7 | Mouse brain | [6] |
| Qx_Limb_Muscle | 3909 | 23341 | 6 | Mouse lower limb muscle | [7] |
| Puram | 5902 | 23686 | 10 | human tissues | [8] |
| Baron-human | 8569 | 20125 | 14 | Human pancreas | [4] |
| Spleen | 9552 | 23341 | 5 | Mouse spleen | [7] |
| Sanderson | 12648 | 16349 | 11 | Mouse tissues | [9] |
| Chen | 14437 | 23284 | 47 | Mouse brain | [10] |
| Zilionis | 34558 | 41861 | 9 | Human lung | [11] |

**Table S2.** Clustering performance accessed by CA on 13 scRNA-seq data sets.

| Data set | scDCCA | scDSC | sciPath | Seurat | SC3 | SCCLRR | MLRSCC | K-means | scMCGF |
| --- | --- | --- | --- | --- | --- | --- | --- | --- | --- |
| Darmanis | 0.764 | 0.721 | 0.783 | 0.618 | 0.697 | **0.837** | 0.716 | 0.620 | 0.835 |
| Usoskin | 0.777 | 0.675 | 0.836 | 0.622 | 0.934 | 0.768 | 0.520 | 0.612 | **0.936** |
| Xin | 0.879 | 0.536 | 0.866 | 0.471 | 0.643 | 0.435 | 0.803 | 0.562 | **0.917** |
| Baron-mouse | 0.862 | 0.717 | 0.571 | 0.669 | 0.709 | 0.571 | 0.683 | 0.672 | **0.949** |
| Muraro | 0.930 | 0.783 | 0.799 | 0.582 | 0.833 | 0.907 | 0.839 | 0.641 | **0.953** |
| Romanov | 0.776 | 0.751 | 0.724 | 0.459 | 0.726 | 0.787 | 0.557 | 0.751 | **0.886** |
| 10X_Muscle | 0.735 | 0.836 | 0.901 | 0.568 | 0.838 | 0.984 | 0.887 | 0.867 | **0.994** |
| Puram | 0.694 | 0.501 | 0.682 | 0.361 | 0.579 | 0.545 | **0.858** | 0.522 | 0.817 |
| Baron-human | **0.873** | 0.653 | 0.657 | 0.554 | 0.589 | 0.468 | 0.820 | 0.611 | 0.863 |
| Spleen | 0.617 | 0.934 | 0.963 | 0.430 | 0.731 | 0.980 | 0.520 | 0.900 | **0.982** |
| Sanderson | 0.677 | 0.504 | 0.614 | 0.298 | 0.339 | 0.430 | 0.555 | 0.345 | **0.972** |
| Chen | 0.703 | 0.644 | 0.442 | 0.580 | 0.428 | 0.451 | 0.355 | 0.480 | **0.767** |
| Zilionis | 0.789 | 0.711 | NA | 0.481 | 0.810 | NA | NA | 0.649 | **0.892** |

*Note:* 10X_Muscle is the abbreviation of Qx_ Limb_Muscle. The results of sciPath, SCCLRR, and MLRSCC are not obtained on the Zilionis data set because of the time complexity.

**Table S3.** Clustering performance accessed by NMI on 13 scRNA-seq data sets.

| Data set | scDCCA | scDSC | sciPath | Seurat | SC3 | SCCLRR | MLRSCC | K-means | scMCGF |
| --- | --- | --- | --- | --- | --- | --- | --- | --- | --- |
| Darmanis | 0.732 | 0.629 | 0.778 | 0.705 | 0.772 | 0.802 | 0.584 | 0.610 | **0.812** |
| Usoskin | 0.576 | 0.444 | 0.852 | 0.730 | **0.875** | 0.575 | 0.371 | 0.392 | 0.835 |
| Xin | 0.775 | 0.402 | 0.756 | 0.665 | 0.722 | 0.595 | 0.559 | 0.649 | **0.855** |
| Baron-mouse | 0.841 | 0.635 | 0.703 | 0.776 | 0.776 | 0.707 | 0.797 | 0.773 | **0.925** |
| Muraro | 0.873 | 0.727 | 0.828 | 0.725 | 0.830 | 0.849 | 0.776 | 0.714 | **0.885** |
| Romanov | 0.705 | 0.647 | 0.581 | 0.609 | 0.527 | 0.680 | 0.547 | 0.632 | **0.718** |
| 10X_Muscle | 0.793 | 0.832 | 0.924 | 0.766 | 0.881 | 0.961 | 0.906 | 0.887 | **0.977** |
| Puram | 0.750 | 0.447 | 0.772 | 0.628 | 0.604 | 0.637 | 0.748 | 0.690 | **0.831** |
| Baron-human | 0.842 | 0.614 | 0.788 | 0.765 | 0.682 | 0.644 | 0.815 | 0.779 | **0.871** |
| Spleen | 0.500 | 0.689 | 0.871 | 0.501 | 0.612 | 0.885 | 0.530 | 0.735 | **0.898** |
| Sanderson | 0.428 | 0.377 | 0.551 | 0.411 | 0.294 | 0.424 | 0.493 | 0.382 | **0.850** |
| Chen | 0.717 | 0.683 | 0.653 | 0.686 | 0.581 | 0.646 | 0.626 | 0.666 | **0.767** |
| Zilionis | 0.826 | 0.616 | NA | 0.682 | 0.778 | NA | NA | 0.668 | **0.852** |

*Note:* 10X_Muscle is the abbreviation of Qx_ Limb_Muscle. The results of sciPath, SCCLRR, and MLRSCC are not obtained on the Zilionis data set because of the time complexity.

**Table S4.** Clustering performance accessed by ARI on 13 scRNA-seq data sets.

| Data set | scDCCA | scDSC | sciPath | Seurat | SC3 | SCCLRR | MLRSCC | K-means | scMCGF |
| --- | --- | --- | --- | --- | --- | --- | --- | --- | --- |
| Darmanis | 0.709 | 0.587 | 0.698 | 0.550 | 0.678 | 0.775 | 0.467 | 0.471 | **0.770** |
| Usoskin | 0.598 | 0.402 | 0.835 | 0.557 | **0.883** | 0.638 | 0.261 | 0.300 | 0.876 |
| Xin | 0.861 | 0.331 | 0.820 | 0.407 | 0.539 | 0.354 | 0.457 | 0.486 | **0.954** |
| Baron-mouse | 0.846 | 0.444 | 0.443 | 0.516 | 0.516 | 0.410 | 0.741 | 0.571 | **0.956** |
| Muraro | 0.914 | 0.533 | 0.701 | 0.436 | 0.735 | 0.830 | 0.772 | 0.571 | **0.918** |
| Romanov | 0.686 | 0.636 | 0.570 | 0.334 | 0.592 | 0.649 | 0.485 | 0.612 | **0.762** |
| 10X_Muscle | 0.690 | 0.818 | 0.915 | 0.466 | 0.831 | 0.964 | 0.876 | 0.864 | **0.988** |
| Puram | 0.670 | 0.352 | 0.612 | 0.273 | 0.467 | 0.449 | 0.644 | 0.453 | **0.715** |
| Baron-human | 0.867 | 0.492 | 0.629 | 0.512 | 0.502 | 0.432 | 0.804 | 0.575 | **0.905** |
| Spleen | 0.368 | 0.824 | 0.942 | 0.213 | 0.523 | 0.941 | 0.294 | 2.789 | **0.957** |
| Sanderson | 0.415 | 0.202 | 0.292 | 0.075 | 0.126 | 0.142 | 0.323 | 0.111 | **0.903** |
| Chen | 0.693 | 0.201 | 0.410 | 0.486 | 0.349 | 0.358 | 0.510 | 0.407 | **0.737** |
| Zilionis | 0.831 | 0.521 | NA | 0.376 | 0.755 | NA | NA | 0.540 | **0.846** |

*Note:* 10X_Muscle is the abbreviation of Qx_ Limb_Muscle. The results of sciPath, SCCLRR, and MLRSCC are not obtained on the Zilionis data set because of the time complexity.

**Table S5.** ARI values of scMCGF on 12 data sets with different parameter k.

(A) Six data sets with less than 3000 cells.

| Data set | k=2 | k=3 | k=4 | k=5 | k=6 | k=7 | k=8 | k=9 | k=10 |
| --- | --- | --- | --- | --- | --- | --- | --- | --- | --- |
| Darmanis | 0.692 | 0.756 | 0.714 | 0.770 | 0.762 | 0.757 | 0.754 | 0.726 | 0.755 |
| Usoskin | 0.685 | 0.448 | 0.859 | 0.876 | 0.878 | 0.879 | 0.859 | 0.881 | 0.915 |
| Xin | 0.955 | 0.956 | 0.954 | 0.954 | 0.932 | 0.958 | 0.961 | 0.952 | 0.949 |
| Baron-mouse | 0.955 | 0.950 | 0.950 | 0.956 | 0.955 | 0.918 | 0.918 | 0.919 | 0.869 |
| Muraro | 0.916 | 0.818 | 0.821 | 0.918 | 0.917 | 0.916 | 0.913 | 0.913 | 0.910 |
| Romanov | 0.303 | 0.309 | 0.260 | 0.762 | 0.764 | 0.766 | 0.686 | 0.765 | 0.763 |

(B) Six data sets with more than 3000 cells.

| Data set | k=11 | k=12 | k=13 | k=14 | k=15 | k=16 | k=17 | k=18 | k=19 | k=20 |
| --- | --- | --- | --- | --- | --- | --- | --- | --- | --- | --- |
| 10X_Muscle | 0.989 | 0.986 | 0.984 | 0.988 | 0.988 | 0.988 | 0.989 | 0.989 | 0.989 | 0.989 |
| Puram | 0.476 | 0.480 | 0.478 | 0.481 | 0.715 | 0.478 | 0.715 | 0.715 | 0.730 | 0.716 |
| Baron-human | 0.905 | 0.613 | 0.613 | 0.627 | 0.905 | 0.799 | 0.781 | 0.615 | 0.638 | 0.614 |
| Spleen | 0.946 | 0.961 | 0.961 | 0.947 | 0.957 | 0.962 | 0.955 | 0.942 | 0.958 | 0.963 |
| Sanderson | 0.834 | 0.843 | 0.845 | 0.828 | 0.903 | 0.902 | 0.843 | 0.905 | 0.906 | 0.853 |
| Chen | 0.712 | 0.682 | 0.724 | 0.711 | 0.714 | 0.720 | 0.717 | 0.720 | 0.716 | 0.717 |

*Note:* 10X_Muscle is the abbreviation of Qx_ Limb_Muscle.

**Table S6.** Performance comparison of scMGCF and its four variants.

| Index | Methods | Darmanis | Romanov | Baron-human | Sanderson |
| --- | --- | --- | --- | --- | --- |
| CA | scMCGF | 0.835 | **0.886** | **0.863** | **0.972** |
|  | scMCGF-pathway | 0.749 | 0.621 | 0.748 | 0.923 |
|  | scMCGF-RNA | 0.813 | 0.841 | 0.596 | 0.949 |
|  | scMCGF-PCA | 0.822 | 0.855 | 0.732 | 0.938 |
|  | scMCGF-DM | **0.841** | 0.847 | 0.747 | 0.918 |
| NMI | scMCGF | 0.813 | **0.718** | **0.871** | **0.850** |
|  | scMCGF-pathway | 0.704 | 0.599 | 0.815 | 0.772 |
|  | scMCGF-RNA | 0.774 | 0.648 | 0.748 | 0.795 |
|  | scMCGF-PCA | 0.779 | 0.664 | 0.813 | 0.819 |
|  | scMCGF-DM | **0.819** | 0.660 | 0.851 | 0.778 |
| ARI | scMCGF | 0.770 | **0.762** | **0.905** | **0.903** |
|  | scMCGF-pathway | 0.610 | 0.418 | 0.761 | 0.834 |
|  | scMCGF-RNA | 0.704 | 0.664 | 0.532 | 0.836 |
|  | scMCGF-PCA | 0.712 | 0.701 | 0.738 | 0.895 |
|  | scMCGF-DM | **0.781** | 0.727 | 0.777 | 0.827 |

**Table S7.** Clustering performance of scMCGF on whole data and down-sampling data.

| Dataset | Index | down-sample  100% data | down-sample  80% data | down-sample  60% data | down-sample  40% data |
| --- | --- | --- | --- | --- | --- |
| Darmanis | CA | 0.835 | 0.783 | 0.754 | 0.866 |
|  | NMI | 0.813 | 0.744 | 0.760 | 0.831 |
|  | ARI | 0.770 | 0.708 | 0.690 | 0.758 |
| Xin | CA | 0.918 | 0.908 | 0.927 | 0.928 |
|  | NMI | 0.855 | 0.807 | 0.804 | 0.834 |
|  | ARI | 0.954 | 0.914 | 0.890 | 0.920 |
| Muraro | CA | 0.953 | 0.948 | 0.943 | 0.919 |
|  | NMI | 0.886 | 0.880 | 0.871 | 0.873 |
|  | ARI | 0.918 | 0.916 | 0.914 | 0.924 |
| Qx_Limb_muscle | CA | 0.994 | 0.994 | 0.997 | 0.996 |
|  | NMI | 0.977 | 0.977 | 0.988 | 0.983 |
|  | ARI | 0.988 | 0.986 | 0.995 | 0.992 |
| Puram | CA | 0.817 | 0.833 | 0.846 | 0.793 |
|  | NMI | 0.831 | 0.832 | 0.844 | 0.799 |
|  | ARI | 0.715 | 0.838 | 0.846 | 0.742 |
| Sanderson | CA | 0.972 | 0.926 | 0.938 | 0.949 |
|  | NMI | 0.850 | 0.821 | 0.850 | 0.829 |
|  | ARI | 0.903 | 0.876 | 0.920 | 0.849 |

[1]. Darmanis S., Sloan S. A., Zhang Y., et al. A survey of human brain transcriptome diversity at the single cell level[J]. Proceedings of the National Academy of Sciences, 2015, 112(23): 7285-7290.

[2]. Usoskin D., Furlan A., Islam S., et al. Unbiased classification of sensory neuron types by large-scale single-cell RNA sequencing[J]. Nature Neuroscience, 2015, 18(1): 145-153.

[3]. Xin Y., Kim J., Okamoto H., et al. RNA sequencing of single human islet cells reveals type 2 diabetes genes[J]. Cell Metabolism, 2016, 24(4): 608-615.

[4]. Baron M., Veres A., Wolock S. L., et al. A single-cell transcriptomic map of the human and mouse pancreas reveals inter-and intra-cell population structure[J]. Cell Systems, 2016, 3(4): 346-360.

[5]. Muraro MJ, Dharmadhikari G, Grün D et al. A single-cell transcriptome atlas of the human pancreas, Cell systems 2016;3:385-394. e383.

[6]. Romanov R. A., Zeisel A., Bakker J., et al. Molecular interrogation of hypothalamic organization reveals distinct dopamine neuronal subtypes[J]. Nature Neuroscience, 2017, 20(2): 176-188.

[7]. Schaum N., Karkanias J., Neff N. F., et al. Single-cell transcriptomics of 20 mouse organs creates a Tabula Muris: The Tabula Muris Consortium[J]. Nature, 2018, 562(7727): 367.

[8]. Puram S. V., Tirosh I., Parikh A. S., et al. Single-cell transcriptomic analysis of primary and metastatic tumor ecosystems in head and neck cancer[J]. Cell, 2017, 171(7): 1611-1624.

[9]. Sanderson S. M., Xiao Z., Wisdom A. J., et al. The Na+/K+ atpase regulates glycolysis and defines immunometabolism in tumors [J]. bioRxiv, 2020: 2020.03. 31.018739.

[10]. Chen R., Wu X., Jiang L., et al. Single-cell RNA-seq reveals hypothalamic cell diversity[J]. Cell Reports, 2017, 18(13): 3227-3241.

[11]. Zilionis R., Engblom C., Pfirschke C., et al. Single-cell transcriptomics of human and mouse lung cancers reveals conserved myeloid populations across individuals and species[J]. Immunity, 2019, 50(5): 1317-1334.
